# Deep Learning for Industrial Computer Vision Quality Control in the Printing Industry 4.0

**DOI:** 10.3390/s19183987

**Published:** 2019-09-15

**Authors:** Javier Villalba-Diez, Daniel Schmidt, Roman Gevers, Joaquín Ordieres-Meré, Martin Buchwitz, Wanja Wellbrock

**Affiliations:** 1 Hochschule Heilbronn, Fakultät Management und Vertrieb, Campus Schwäbisch Hall, 74523 Schwäbisch Hall, Germany; Wanja.wellbrock@hs-heilbronn.de; 2 Department of Artificial Intelligence, Escuela Técnica Superior de Ingenieros Informáticos, Universidad Politécnica de Madrid, 28660 Madrid, Spain; 3 Matthews International GmbH, Gutenbergstraße 1-3, 48691 Vreden, Germany; Daniel.Schmidt@saueressig.de (D.S.); roman.gevers@saueressig.de (R.G.); 4 Departament of Business Intelligence, Escuela Técnica Superior de Ingenieros Industriales, Universidad Politécnica de Madrid, 28006 Madrid, Spain; j.ordieres@upm.es; 5 InspectOnline, Wiley-VCH Verlag GmbH & Co. KGaA, 69469 Weinheim, Germany; mbuchwitz@wiley.com

**Keywords:** soft sensors, industrial optical quality inspection, deep learning, artificial vision

## Abstract

Rapid and accurate industrial inspection to ensure the highest quality standards at a competitive price is one of the biggest challenges in the manufacturing industry. This paper shows an application of how a Deep Learning soft sensor application can be combined with a high-resolution optical quality control camera to increase the accuracy and reduce the cost of an industrial visual inspection process in the Printing Industry 4.0. During the process of producing gravure cylinders, mistakes like holes in the printing cylinder are inevitable. In order to improve the defect detection performance and reduce quality inspection costs by process automation, this paper proposes a deep neural network (DNN) soft sensor that compares the scanned surface to the used engraving file and performs an automatic quality control process by learning features through exposure to training data. The DNN sensor developed achieved a fully ***automated classification accuracy rate of 98.4%***. Further research aims to use these results to three ends. Firstly, to predict the amount of errors a cylinder has, to further support the human operation by showing the error probability to the operator, and finally to decide autonomously about product quality without human involvement.

## 1. Introduction

Countries aspiring to lead these technological changes and remain in industrial leadership positions have strategically positioned themselves for the new type of cyber–physical infrastructure that will emerge from the Industrial Internet of Things (IIoT) and data science. Germany’s Industry 4.0 framework has evolved into a pan-European collaborative effort to perform intelligent automation at scale [1]. In a similar move, the United States launched the Manufacturing Leadership Coalition (SMLC) [2] in 2011. Other notable examples include “China Manufacturing 2025” [3] that seeks to elevate advanced manufacturing technology, or Japanese’s “Society 5.0” [4] with a holistic focus on the safety and well-being of humans through cyber–physical systems. As a paradigmatic example, the Japanese manufacturer has consistently gained a competitive edge towards its competition by providing its value stream elements with the ability not to pass defects to the next step in the manufacturing process [5].

A prime example of this is the remarkable success of Toyota’s implementation of intelligent autonomation, or JIDOKA -UTF8min自働化- [6,7,8], alongside other strategic Lean manufacturing system characteristics [9,10,11,12,13,14]. Thanks to the availability of sufficient data from virtually any element of the production process (through IIoT for example), and the development of computational elements powerful enough to perform real time calculations on the state of the value stream, the systematic extension of JIDOKA in the industry has been made possible [15]. In fact, there is great potential for other industries to increase the ability of machines to recognize their own state through intelligent sensors capable of ***sensing*** the specific needs of customers and ***responding*** flexibly and accordingly. This would improve the level of automation and increase product quality and customization while increasing related value stream performance [16,17,18].

Within this framework, Optical Quality Control (OQC) is crucial to many manufacturing processes in an effort to meet customer requirements [19]. On the one hand, the performance of human-centered OQC does not meet the necessary requirements: it is limited by ergonomics and cost, as humans get tired with repetitive OQC tasks and these tasks are usually very labor-intensive. For this reason, automatic detection of visual defects, which aims to segment possible defective areas of a product image and subsequently classify them into defect categories, emerges as a necessary solution to the problem. On the other hand, simple threshold techniques are often insufficient to segment background defects when not applied to a controlled environment characterized by stable lighting conditions. Xie [20] provides a classification of existing methods, but the common practice in industrial environments is that each new feature has to be described manually by experts when a new type of problem occurs: surface defects in industrially manufactured products can have all kinds of sizes, shapes or orientations. These methods are often not valid when applied to real surfaces with rough textures, complex, or noisy sensor data. This has the immediate consequence that classifications are almost always insufficient and cannot be generalized to unknown problems [21]. For these reasons, more robust and reliable results are needed in the detection of defects by more sophisticated methods.

The printing industry underwent an enormous transformation through the digital revolution when inkjet reached a mature era. Inkjet printing is based on the formation of small liquid droplets to transfer precise amounts of material to a substrate under digital control. Inkjet technology is becoming relatively mature and is of great industrial interest due to its flexibility for graphic printing and its potential use in less conventional applications such as additive manufacturing and the manufacture of printed electronics and other functional devices. Its advantages over conventional printing processes are numerous. For instance, it produces little or not waste, it versatile thanks to different processes, it is non-contact, and does not require a master template which means printing patterns can be easily changed. However, the technology needs to be developed in order to be used in new applications such as additive manufacturing (3D printing).

**Figure 1:**
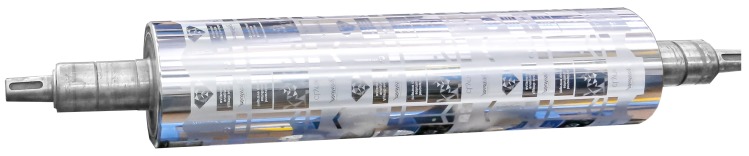
Printing Cylinder.

Laser engraving of gravure cylinders (Figure 1) is the latest and most exciting development in gravure printing. Laser technology makes it possible to produce cells with variable shapes, which is not possible with electromechanical engraving. These new shapes actually provide a higher print density and it is possible to use inks with a higher viscosity than conventional electromechanically engraved cylinders. Laser engraved cylinders also reduce the influence of print speed on print quality and keep the highlight tone values stable.

Although laser engraving of rotogravure cylinders is a new variant of etching rotogravure cylinders in the rotogravure market, today’s systems are still susceptible to errors. Possible errors or optical detectable defects include dents, scratches, inclusions, spray, curves, offset, smearing and excessive, pale or missing printing or color errors (i.e., incorrect colors, gradients and color deviations from the desired pattern). The most common errors is dents, 32%, while the least common error is smearing, 3%. Due to the different errors and noise levels typical of industrial settings, an automatic error detection based on classical computer vision algorithms was not possible [22]. Most systems aim to select potential faults and present them to the human expert responsible for deciding the presence or severity of faults. Practice shows that about 30% of the possible errors that need to be checked are not relevant. This fact increases both the costs associated with the OQC and the lead time of the overall process. Both factors are crucial to achieving customer confidence and must be systematically optimized.

Bearing these issues in mind, this research delves into an alternative solution to overcome the problem of the need of manual predetermination of the specific characteristics for each new inspection problem: deep learning-based deep neural networks (DNN). Deep learning is a paradigm of machine learning that enables computational models consisting of multiple processing layers to learn representations of data with multiple levels of abstraction [23,24]. DNN are constructions created by combining a series of hierarchically superimposed and arbitrarily initialized filters that are capable of automatically learning the best features for a given classification problem due to exposure to training data [25,26]. Several DNN architectures have been successfully used to extract statistical information from physical sensors in the context of Industry 4.0 in several applications such as classification problems [27], visual object recognition [23], human activity recognition through wearables [28,29], predictive maintenance [30,31], or computer vision [32] among others. More specifically, DNN have recently proved useful for industrial computer OQC defect detection purposes with promising results by automatically extracting useful features with little to no prior knowledge about the images [33,34].

The goal of this paper is to present a soft sensor DNN that performs a ***classification*** of images from high-resolution cameras towards a fully computer vision OQC of the printing cylinder of a global leading player in the Printing Industry 4.0. As shown in detail in Section 3, this aims to increase the accuracy of the quality inspection process by first supporting the human expert final decision making, thereby reducing the cost of quality inspection process through automatization of the visual processing. This ought to be contextualized in a hostile industrial context in which the complexity of error detection is very high due both to the extraordinary variability of possible errors, as well as the changing environmental conditions of light, moisture, dirt, and pollution - all of which can confuse the best algorithms developed thus far.

The rest of the paper is structured to ensure clarity in the presentation, replication of the results obtained, and a proper framing in the ongoing global context of the fourth industrial revolution. Firstly, Section 2 briefly shows the continuous improvement of the manufacturing value stream of an Industry 4.0 leader that made the integration of deep learning technology possible. Secondly, Section 3 outlines the ***materials and methods*** used to design and implement a better performing OQC integrated DNN soft sensor. Additionally, DNN computer Code is made available on an Open Access Repository. Next, the ***results*** obtained are briefly discussed from a technical point of view in Section 4. Finally, in Section 5 the short, medium and long term ***consequences*** of these findings for the printing industry are discussed and highlighted in a broader manufacturing Industry 4.0 context.

## 2. Evolution towards Automatic Deep Learning-Based OQC

In order to frame this research in a more general context and allow its replication in other value streams, it is important to describe the constant process of continuous improvement [35] that a leading player in the printing industry has followed in recent years to reach the level that has allowed the implementation of the presented Deep Learning-based OQC research.

**Figure 2:**
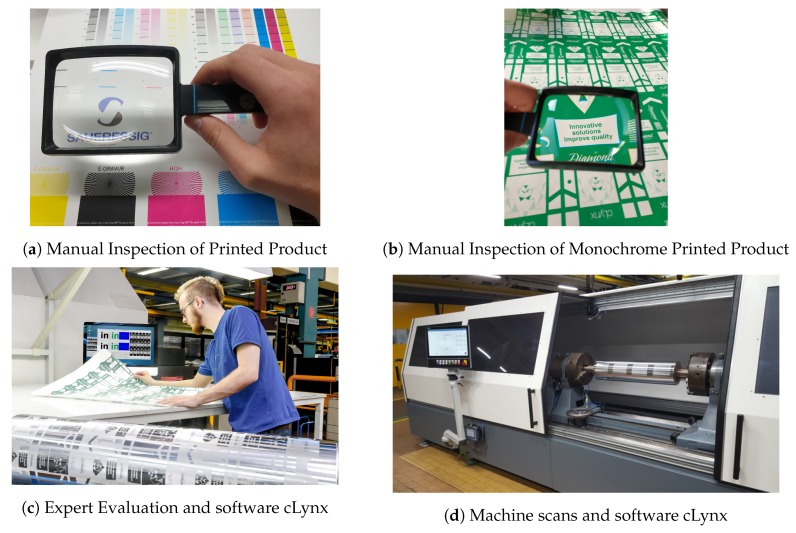
OQC evolutional continuous improvement process.

For the purpose of making it easier for interested readers to recognize the fundamental phases of this OQC evolutional continuous improvement process that paved the road for a fully automatized computer vision OQC process have been summarized in Table 1 and is depicted in Figure 2.

## 3. Deep Learning for Industrial Computer Vision Quality Control

In order to reduce time checking possible mistakes on the cylinder, and further reduce OQC cost and value stream-related lead time, an automatic pre-selection of the errors using artificial intelligence is desired. Due to intensive research investment and strategic focus on quality control throughout the value stream process, real noisy industrial data has been classified and properly labelled. This is how the idea was born to design a DNN that would learn from the statistical information embedded within the previously classified data to perform a fully automated computer vision quality control.

Due to intensive research investment and strategic focus on quality control throughout the value stream process, there were previously numerous classified and properly labeled data aggregated through fourth stage. Possible errors were selected using thresholds between the original file and the scanned cylinder. These were then shown to the operator, who judged them as if they were real errors. These judgements were then saved comprising the labeled data-set.

**Figure 5:**
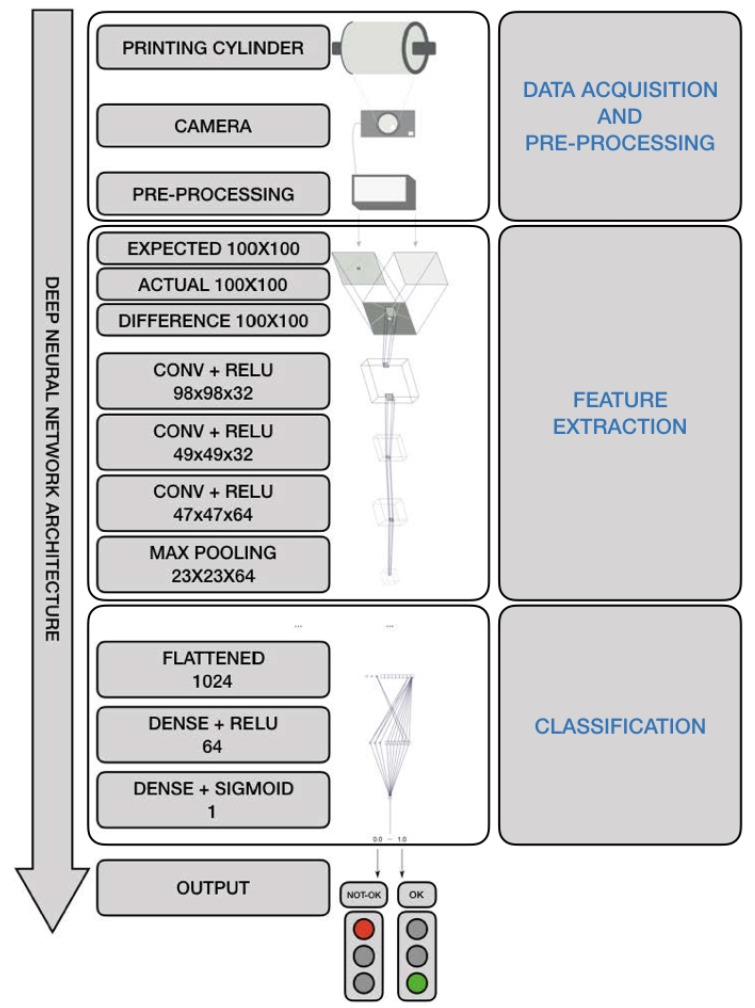
Deep Learning Architecture for Industrial Computer Vision OQC in the Printing Industry 4.0.

In the fifth stage the process is taken over by a fully automated DNN architecture, as shown in Figure 5, and as proposed in this paper (see Section 3.1.3), after an intensive experimental program, which has tested different architectures (DNN, restricted boltzmann machines, deep belief networks, etc.) and configurations of different filter sizes, abstraction layers, etc. [37].

***The DNN soft sensor presented achieves an accuracy of 98.4% in fully automatic recognition of production errors.*** More details are provided in the following subsections. This contribution makes it possible to decide immediately after scanning whether the cylinder can be delivered or whether errors need to be corrected. It was decided not to use specific denoising treatments as specific filters before classification [38,39]. This is because of the intrinsic capabilities found in the adopted CNN architecture.

### 3.1. Deep Neural Network Architecture for Computer Vision in Industrial Quality Control in the Printing Industry 4.0

#### 3.1.1. Experimental Setup

The experiments in this study were implemented with a computer equipped with an Intel(R) Xeon(R) Gold 6154 3.00GHz CPU and an NVIDIA Quadro P4000 Graphic Process Unit (GPU) with 96 GB of random-access memory (RAM). The operating system was ***Red Hat Linux*** 16.04 64-bit version.

The deep learning model training and testing were conducted with ***Keras*** which is an interface for ***TensorFlow*** (Version 1.8), and the model was built in ***Python*** (Version 2.7) language [40]. TensorFlow is an interface for expressing machine learning algorithms, and an application for executing such algorithms, including training and inference algorithms for DNN models. More specifically, the TF.Learn module of TensorFlow was adopted for creating, configuring, training, and evaluating the DNN. TF.Learn is a high-level Python module for distributed machine learning inside TensorFlow. It integrates a wide range of state-of-the-art machine learning algorithms built on top of TensorFlow’s low-level APIs for small- to large-scale supervised and unsupervised problems. Additional Python interfaces were used: ***OpenCV*** for computer vision algorithms and image processing, ***Numpy*** for scientific computing and array calculation, and ***Matplotlib*** for displaying plots. The details of building the DNN model for OQC with Python are provided online at Open Access Repository and were created with ***Jupyter Notebook***.

#### 3.1.2. Data Pre-Processing

In order to train the DNN, standardized classified input data is needed. For this reason, the Data pre-processing is divided in three steps: (1) decision of which is the size of the image that serves as input for the DNN and what the size of the convolutional window used by the DNN should be, (2) brightness adjustment through a histogram stretching, and (3) automatize the selection and labelling of the file structure to be fed to the DNN.

**Figure 6:**
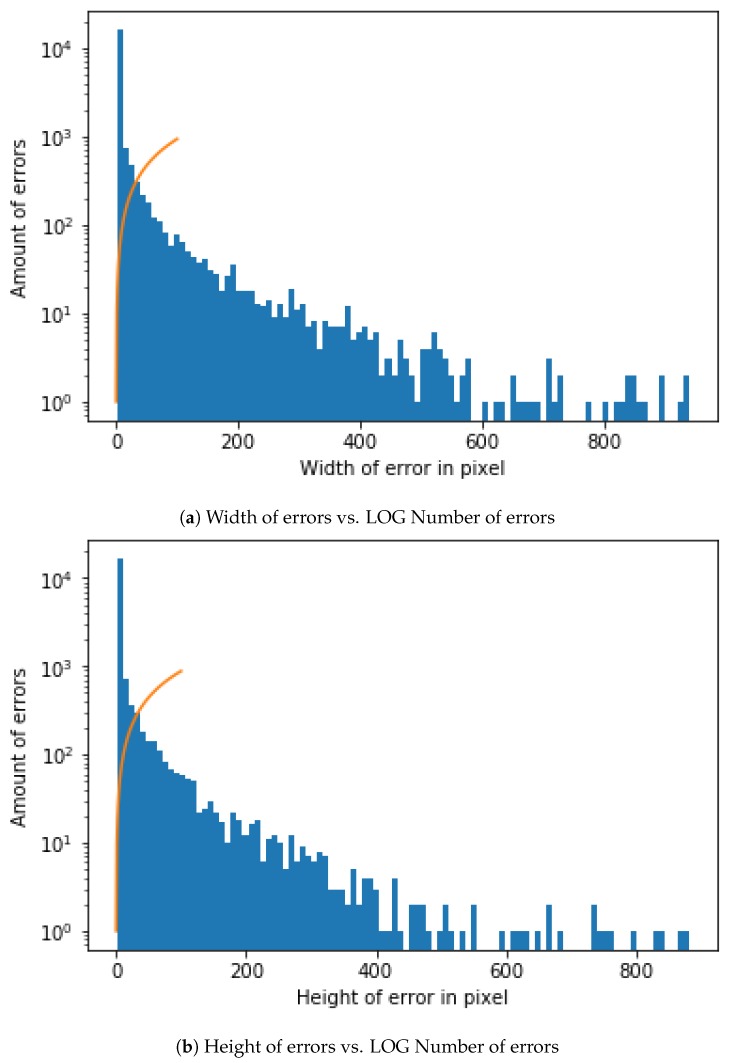
Aspect Ratio Inspection.

**Figure 7:**
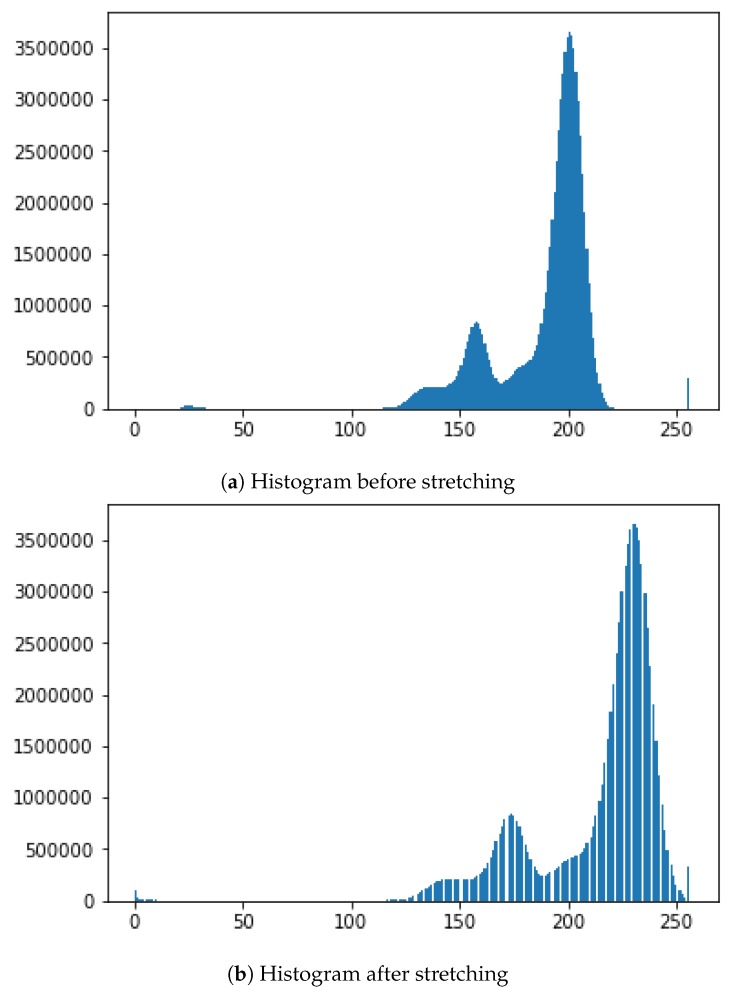
Pre-processing Histogram for brightness adjustment.

**Figure 8:**
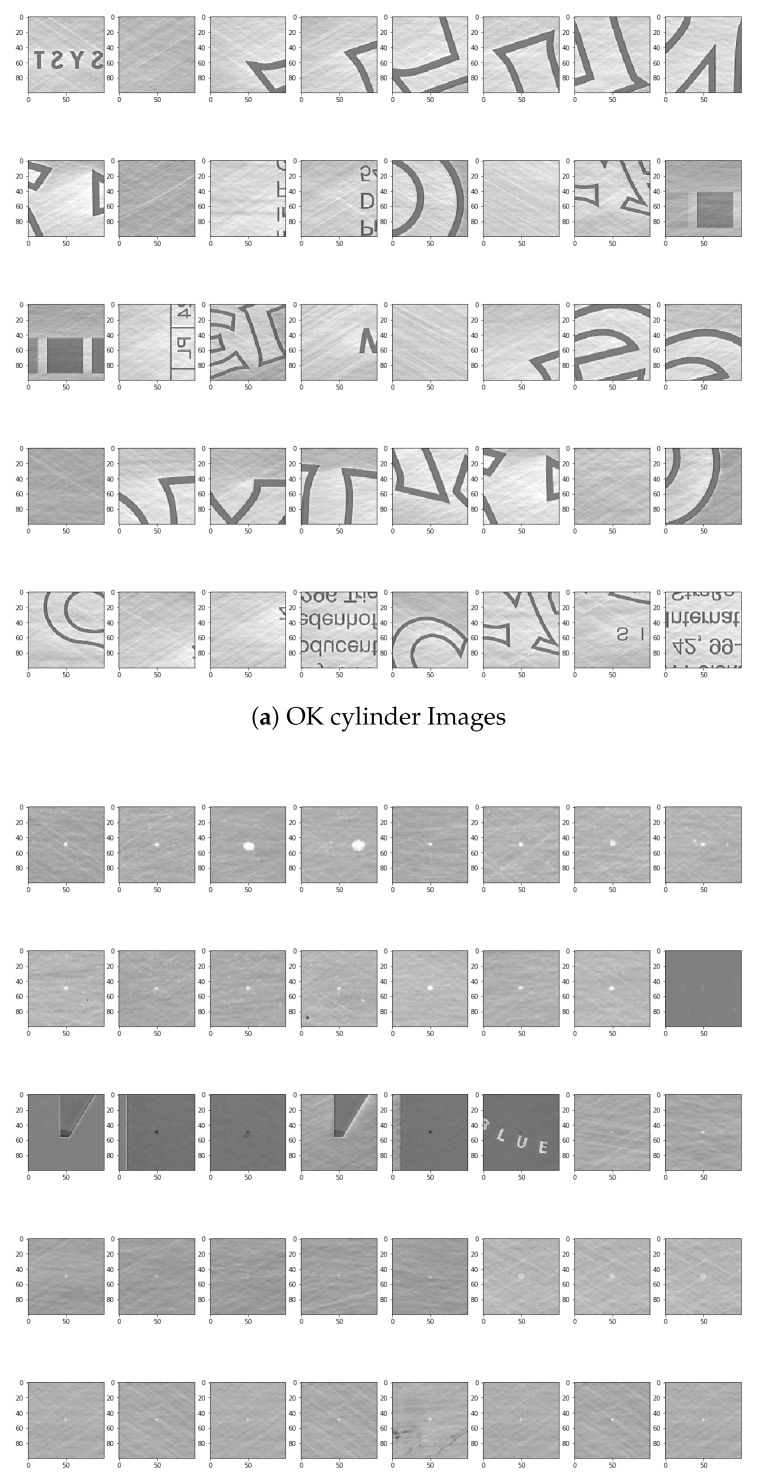
Examples of OK cylinder and not-OK cylinder Images.

1. Image Size for DNN Input and Convolutional Window Size
  Due to the need for standardized input data, a decision needs to be made about which dimensions the input images should have. The first decision is the aspect ratio. The following decision should be how many pixels wide and high the input images should be. In order to get a first impression of the existing sizes, a short analysis of the previous manually confirmed errors is made. According to the data, the mean value of the width is slightly higher than that of the height. In the mean aspect ratio this gets even clearer with a mean aspect ratio of about 1.5. This is probably a result of some errors that are elongated by the rotation of the cylinder. The median aspect ratio is exactly at 1.0. Because the median describes a higher percentage of errors better this should also be the aspect ratio of the neural network input. As shown in the representation of the width and height of error in pixel against the LOG of the amount of errors Figure 6.
  As the size of the error also plays a role in the judgment of the errors, scaling operations should be reduced to a minimum. Due to the range of the sizes this is not always possible. The training time of the neural network would increase dramatically with large input sizes and small errors would mostly consist of ***OK***-cylinder surface. Therefore a middle ground is needed so that most input images can be shown without much scaling or added ***OK***-cylinder surface. A size in the middle would be 100 pixels. We therefore calculate the percentage of errors with the width smaller or equal to 100. The results show that about 90% of all errors have both the height and width below or equal to 100 and almost 74% have both the height and width below or equal to 10. One option would be to use an input size of 100 × 100.
2. Brightness Adjustment
  To get comparable data for all cylinder images, pre-processing is needed and is performed on the complete scan of a cylinder. From this scan multiple examples are taken. Because there can be slight deviations due to many influences during the recording of the cylinder surface, this can only be achieved by having a similar brightness for the cylinder surface and engraved parts. Another important point is that no essential information gets lost from the images and, that the brightness between the engraved and not engraved parts are comparable for all cylinder scans. Therefore a brightness stretch is needed but only few pixels are allowed to become the darkest or brightest pixels. Notwithstanding, the amount of pixel that become the darkest and brightest pixels ca not be set to a very low value because noise in the image data would result in big differences. In conclusion a low percentage of the pixels should be set as darkest and brightest. For example, the lowest and the highest percentage should each have a maximum of 0.5%. Figure 7 shows a stretching example for brightness adjustment for one image so that 0.5% of all pixels will have a value of 0 and 0.5% of all pixels will have the value of 255.
3. Automatic selection and Dataset Labelling
  To simplify the later steps, the images need to be cut from the original file and saved into two folders with examples that are ***OK***-cylinder (Figure 8a) and examples that are ***not-OK***-cylinder (Figure 8b). The great variety of patterns presented in the spectrum can be observed in the figures. The very nature of the process implies that each new product represents a new challenge for DNN, as it has probably never before been confronted with these images. For this reason, the errors may be of a very different nature. This implies a high complexity of solving the challenge of training and testing the DNN. Likewise, the different shades of black and grey, very difficult to appreciate with the naked eye when manually sorting the images, represent an added difficulty that must be resolved by DNN architecture.
  If errors are smaller in width or height than 100, the ROI gets increased to 100. If any size is bigger than 100 pixels is ignored. For the purpose of checking later on, the big input data is split into 100 × 100 parts. If any one of these is detected as an error, all are marked as an error. As shown in the Open Access Repository, there are multiple possible ways to handle the bigger data. Every example also has the actual and target data. There are different ways of using this data as input. One way is just using the actual data. A different option is to use the difference between the actual and expected data. The problem in both cases is that information gets lost. Better results have been achieved by using the differences. These get adjusted, so that the input data is in a range from [−1,1]. Once this is performed, and because a balanced dataset is important to train the neural network and the ***OK***-cylinder examples far outnumber the ***not-OK***-cylinder examples, an ***OK***-cylinder example is only saved if a ***not-OK***-cylinder example has been found previously.

#### 3.1.3. Automatic Detection of Cylinder ErrorsUsing a DNN Soft Sensor

The DNN soft sensor architecture design is performed with two main goals in mind: classification and performance:

- *Classification* The first goal of this architecture is not to identify different objects inside of part of the images but to separate two classes (***not-OK*** and ***OK*** images), where the main source of noise came from the illumination factor from the scanner lectures. Therefore, neither the so deep architectures nor the identity transference, which was the key for the ResNet [41] is needed in our case, and just few convolutions shall help identify convenient structural features to rely on.
- *Performance*. The proposed architecture is even more simplistic than the AlexNet [42] one, as we do not use five convolution layers but just three. The main reason is to look for a compromise between the number of parameters and the available dataset of images. Our architecture was always looking to be *frugal* in terms of resources, as it is expected to be a soft sensor, running in real time and having the inherent capability of retrain for reinforced learning, close to such real time constraint.

After data acquisition and pre-processing, the input data of the DNN are figures represented as tensors. A type of network that performs well on the classification problem of such data is usually divided in two main parts: feature extractors and classifiers as shown in Figure 5:

- *Feature Extraction*. The feature extraction is performed by a deep stack of alternatively fully connected convolutional and sub-sampling max pooling layers, the even numbered layers are for convolutions and the odd numbered layers are for max-pooling operations.
  - *Convolution and ReLu (rectified linear unit) activated convolutional layers*. Convolution operations, by means of activation functions, extract the features from the input information which are propagated to deeper level layers. A *ReLu* activation function is a function meant to zero out negative values. The *ReLu* activation function was first presented in AlexNet [42] and solves the vanishing gradient problem for training DNN.
  - *Max pooling*. Consists of extracting windows from the input feature maps and outputting the max value of each channel. It’s conceptually similar to convolution, except that instead of transforming local patches via a learned linear transformation (the convolution kernel), they are transformed via a max tensor operation.
- *Classification*. The classification is performed by fully connected activation layers [43]. Some examples of such models are LeNet [44], AlexNet [42], Network in Network [45], GoogLeNet [46,47,48], DenseNet [49].
  - Fully connected activation layers output a probability distribution over the output classes [25]. Because we are facing a binary classification problem and the output of our network is a probability, it is best to use the binary-crossentropy loss function. Crossentropy is a quantity from the field of Information Theory that measures the distance between probability distributions or, in this case, between the ground-truth distribution and the predictions. It is not the only viable choice: we could use, for instance, mean-squared-error. However, crossentropy is usually the best choice when dealing with models that output probabilities. Because we are *attacking* a binary-classification problem, we end the network with a single unit (a Dense layer of size 1) and a sigmoid activation. This unit will encode the probability that the network is looking at one class or the other [25].

**Figure 9:**
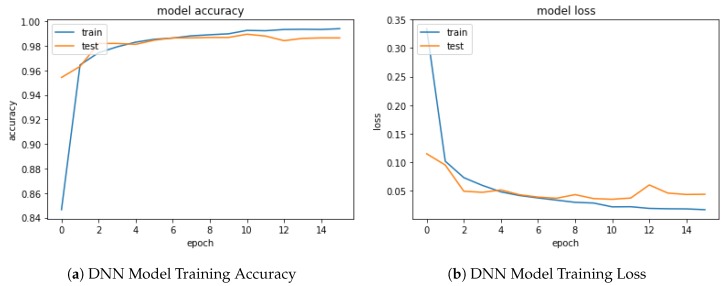
DNN Training and Testing Results.

As shown in the Open Access Repository, using Keras, Tensorflow backend for the DNN and OpenCV/Numpy for the image manipulation, a balanced dataset of 13,335 ***not-OK***- and 13335 ***OK***-cylinder examples is used, giving a total of 26,670. These were collected over a period of 14 months from almost 4000 cylinder scans. The training part is mirrored vertically and horizontally resulting in 85,344 training samples in total. All ***not-OK***- cylinder examples are labeled ***0*** and all Ok examples are labeled ***1***. As a standard procedure, the data is split into ***training dataset*** (80%), ***testing dataset*** (10%) and ***validation dataset*** (10%). The ***training dataset*** is used to train the DNN throughout an number of epochs as shown in Figure 9. It can be observed that both accuracy and loss do not increase or decrease significantly after epoch number 10.

**Figure 10:**
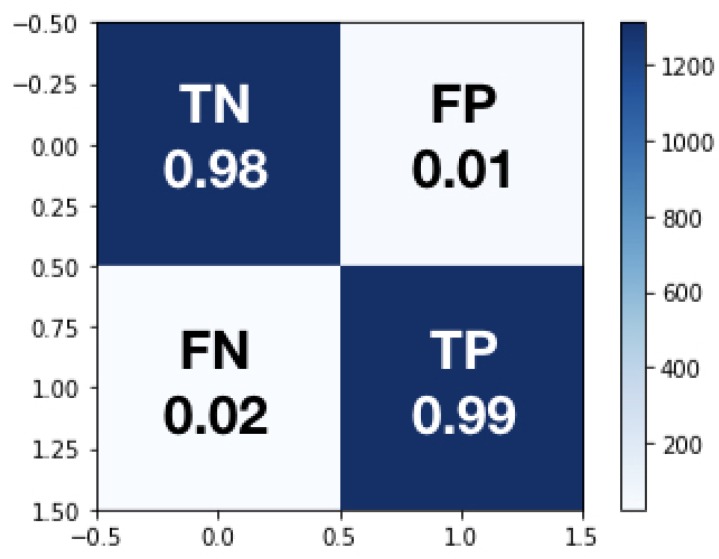
DNN Model Testing Confusion Matrix.

The ***testing dataset*** is subsequently used to test DNN performance. The confusion matrix is a standard procedure to summarize the results of such a training by typically combining contingency classes (*TRUE*, *FALSE*) and (*OK*, *not-OK*), hence building four categories: (1) True Negative (*TN*), which is an error and has been predicted as an error; (2) False Positive (*FP*), which is an error but has not been predicted as an error, and is by far the most damaging category; False Negative (*FN*) which is not an error but has been predicted as an error; and (4), True Positive (*TP*) which is not an error and has not been predicted as an error. Specifically, given the balanced dataset chosen, the accuracy (ACC) delivered by the DNN soft sensor, defined by the expression ACC=(TP+TN)/(TP+TN+FP+FN), is 98.4%. The *TN* rate is 97.85%, the *TP* rate is 99.01%, the *FN* rate is 2.15% and the *FP* rate is 0.99%. These levels of *ACC* can be considered acceptable for such a complicated industrial classification problem. The results are summarized in Figure 10.

**Figure 11:**
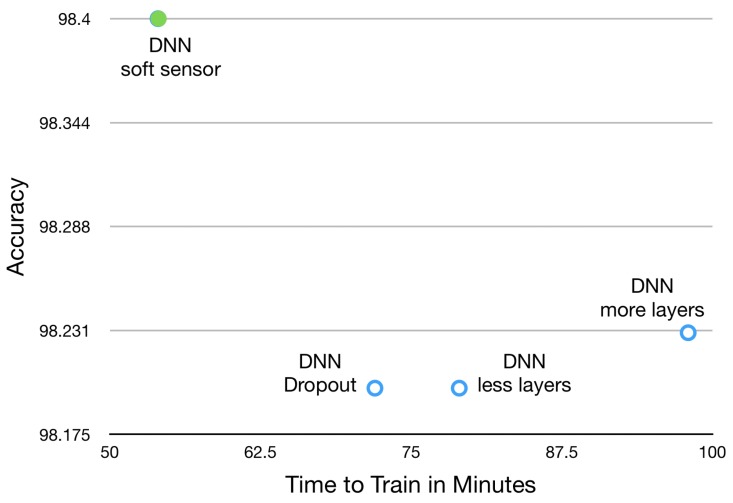
Deep Learning Architecture Comparison. Time to Train vs. Accuracy.

In Table 2 the DNN architecture shown in Figure 5 is described layer by layer by outlining the rationale behind the choice of a layer rather than another. Going even further, to compare the performance of the proposed soft DNN sensor, it has been compared with three similar architectures. The result of this comparison is shown in Open Access Repository and summarized in Figure 11 in which it is clearly shown that the proposed DNN soft sensor has superior performance to other alternative architectures.

Two parameters, accuracy and computational time, have been measured consistently with the same training and test set, and then compared. First, it has been tested with an identical architecture by adding a dropout, then it has been tested with a deeper architecture and finally with a more shallow DNN with fewer layers. The accuracy should be as high as possible in order to generate the lowest possible error in data characterization, and the computation time should be as low as possible in order to ensure that the soft DNN sensor can be effectively integrated into an Industry 4.0 environment, thus ensuring maximum effectiveness and efficiency respectively. A smooth DNN sensor must be not only accurate but also fast to ensure, among other things, a minimum Lead Time impact on the value creation process and low CO${}_{2}$ emissions derived from the energy consumption associated with the computation.

#### 3.1.4. Visualizing the Learned Features

Experience has shown that visualizing what each of the DNN layers learns can help deep architecture designers improve their understanding of the learning of the DNN hidden layers and thus support an appropriate fine tuning of their design for improvement purposes. This is because visualizing what the DNN has learned can help in the understanding of the decision making process. There are different ways of visualizing what has been learned by showing different parts. These can make it easier to understand why some things do not work as expected. For example why some pictures with errors were not categorized as errors (FP).

This visualization can be performed in different ways. For instance, given an example image of a ***not-OK*** cylinder shown in Figure 13a, an option is to visualize what the DNN captures using class activation heatmaps. A class activation heatmap is a 2D grid of scores associated with a specific output class, computed for every location in any input image, indicating how important each location is with respect to the class under consideration. An example is shown in Figure 13b.

Another option is to calculate an input image that gets the highest response from a layer. This is done by displaying the visual pattern that each filter is meant to respond to. This can be done with gradient ascent in input space: applying gradient descent to the value of the input image of a convolutional network so as to maximize the response of a specific filter, starting from a blank input image. The resulting input image will be one that the chosen filter is maximally responsive to. An example is shown in Figure 14.

**Figure 12:**
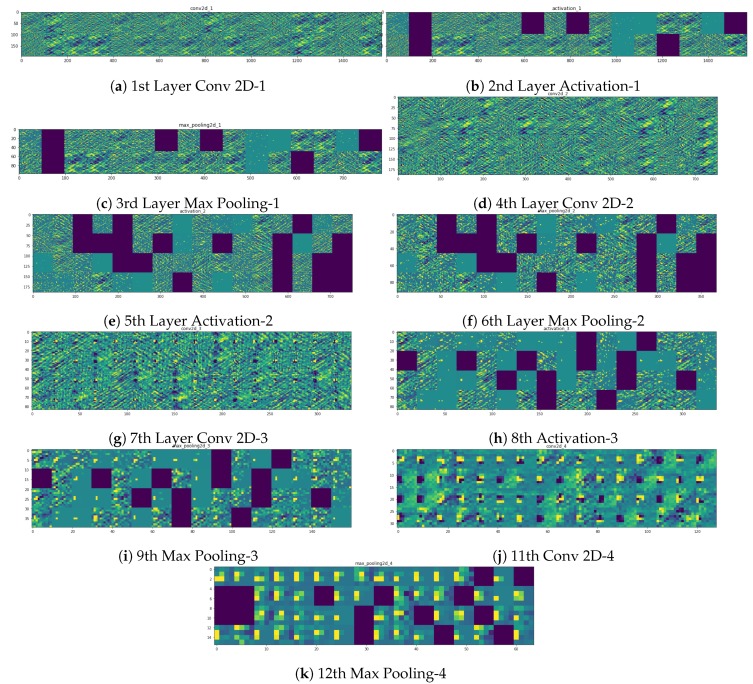
Visualization of all DNN layers as color-coded images of a *TN* image.

**Figure 13:**
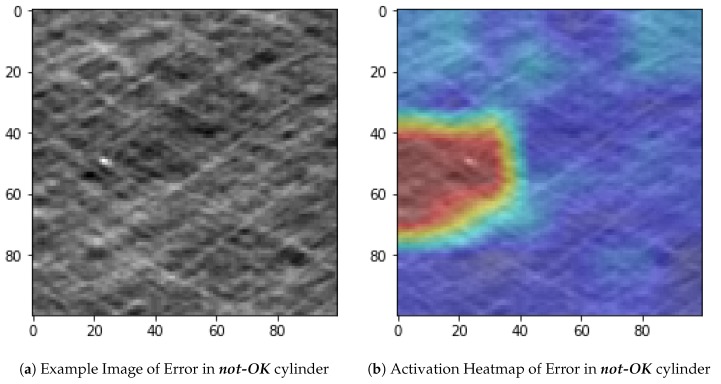
Example Image ***not-OK***-cylinder and Activation Heatmap

**Figure 14:**
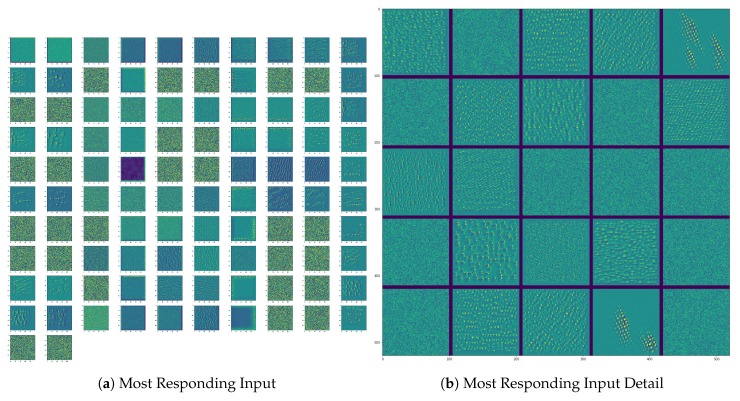
Most Responding Input.

Finally, an alternative approach would be to show the outputs of all DNN layers as color-coded images. Visualizing intermediate activations consists of displaying the feature maps that are output by various convolution and pooling layers in a network, given a certain input (the output of a layer is often called its activation, the output of the activation function). This gives a view into how an input is decomposed into the different filters learned by the network. We want to visualize feature maps with three dimensions: width, height, and depth (channels). Each channel encodes relatively independent features, so the proper way to visualize these feature maps is by independently plotting the contents of every channel as a 2D image. For explanatory purposes, on the Open Access Repository, four different examples, *TP*-*TN*-*FP*-*FN*, of such feature maps are depicted. These shall help the reader better understand what the DNN *sees* and how it *responds* in different circumstances. One of these examples, *TN*, is visualized in Figure 12.

## 4. Results and Discussion

Due to the automation by means of the soft DNN sensor, the costs associated with OQC could be drastically reduced. Also, the accuracy of error detection increased considerably. The results can be therefore considered *very promising* and allow for different ways of further industrial implementation. However, these results have to be interpreted in a broad context of Industry 4.0. This section provides some essential aspects that will help to understand and contextualize the contributed results through a meta-discussion at various organizational levels. This will help to present in the next section a possible future strategic development of these ***deep technologies*** in the short, medium and long term.

There are different steps that have to be taken until the full potential can be used in the production without taking a too high risk of missing an error.

1. Using the DNN fully automate OQC classification to predict the amount of errors a cylinder has.
  The DNN *only* provides a successful result 98.4% of the time. To be sure that the wrongly classified images are not big mistakes, human experts will review all possible errors. DNN has already had a positive influence on the workflow, as we know how many errors are very likely an error: DNN helps significantly in the planning of the next workflow step because it is known with a high probability if the cylinder needs to go to the correction department or if it is very likely that the product is an ***OK***-cylinder.
2. Showing the error probability to the operator that is currently deciding if it is an error or if it is not.
  This gives a hint to the operator, who can give feedback if there are relevant mistakes that were not predicted as mistakes. This can also help the operator to reduce the likelihood of missing an error. Once this soft sensor was integrated in production, OQC productivity, measured in hours per unit - time an operator spends in the OQC -, dramatically increased by ***210%*** as decision about defects is made in an automatic way.
3. Only showing possible errors that have been predicted by the DNN.
  In the last step, the DNN could completely filter out errors that are not relevant. This can also be used in multiple steps because it is possible to increase the threshold error probability for the possible error to be shown. At some point a threshold will have to be chosen taking into consideration the cost of checking a possible error and the cost of missing a error. This would completely eliminate the step of checking the errors and the confirmed errors would only be checked by the correction department.

## 5. Conclusions and Future Steps of Deep Learning in a Printing Industry 4.0 Context

Although there has been an immediate performance increase in OQC error detection accuracy and cost effectiveness, larger scope for improvement is down to the managerial dimension of such a sensor. This is because it can be expanded to not only detect defects but also to classify them in categories. Although this requires additional effort, it will enable the cause-effect analysis regarding manufacturing conditions and defect frequencies.

**Figure 15:**
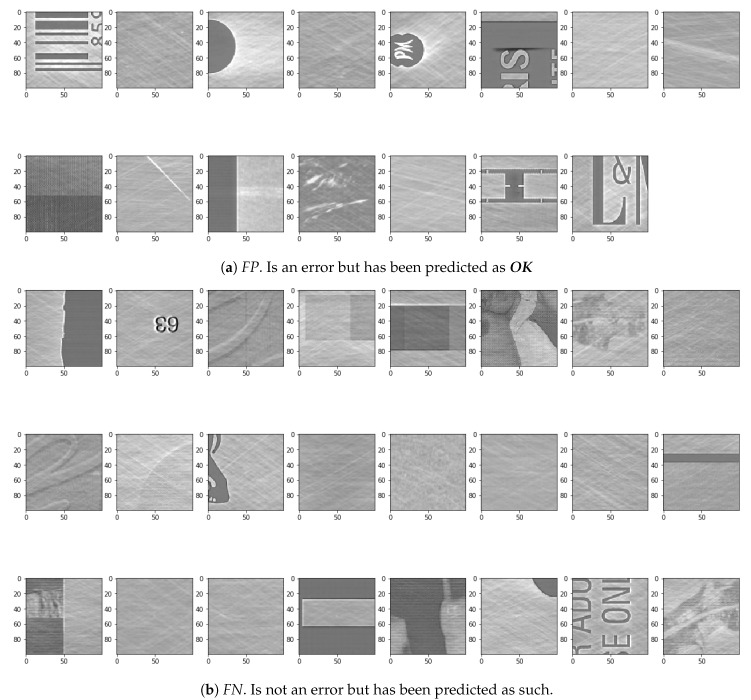
Examples of *FP* and *FN* Images.

Some of these efforts can be specifically targeted to achieve an improvement in the accuracy of the model. For example learning from the false predictions: to further improve the correct prediction rate it is important to take a look at the examples that have not been predicted correctly. This could potentially improve the understanding why the wrong prediction was made by the DNN:

- ***Not-OK**examples that have been predicted as**OK***. Looking at the actual errors in the test data that have not been predicted as errors, as in Figure 15a, a few issues could be the cause of the wrong predictions. Some of the examples actually do not look like they are really ***not-OK***. The cause of this could either be, that the input data was not labeled correctly or that the error really is not highly visible in the image.
- ***OK**examples that have been predicted as**not-OK***. After looking at the visualization of the DNN, it gets clear that the main focus for finding mistakes is looking for extreme edges. These can be seen in a lot of the wrongly classified examples. Especially the first two examples seen in Figure 15b have some extreme edges that are a result of a slight misalignment of the images in the pre-processing. Therefore the image registration in the pre-processing part between the original and the recording of the cylinder surface needs to be improved.

This technology could also be implemented at the customer side to increase defect detection accuracy on the printed product itself. This strategic step is currently being discussed internally. Such analyses will provide sensitivity about operations and operational conditions, which will also impact in value stream-related efficiency and effectiveness.

These aspects will probably be the next steps in further research actions to be developed within an Industry 4.0 context. For instance, deep learning applied to manufacturing Industry 4.0 technology will have an impact at various levels of aggregation in the printing manufacturing value chains:

1. Deep Learning at a shopfloor level shall impact quality, reliability and cost.
  At the shopfloor level, this paper has shown an example of how deep learning increases the effectiveness and efficiency of process control aimed at achieving better quality (e.g., with OQC) and lower costs, allowing self-correction of processes by means of shorter and more accurate quality feedback loops. This intelligence integrated in the value streams will allow many humans and machines to co-exist in a way in which artificial intelligence will complement in many aspects. In the future, significant challenges will still be encountered in the generation and collection of data from the shopfloor.
  The main challenge towards a fully automated solution is currently getting the Python DNN integrated into the C++ cLynx program. After this is successfully completed, a testing phase with the cLynx users is planned. If the results are satisfactory, the complete automatic process will be started. If the results are not satisfying, further steps have to be taken so as to improve the DNN further.
2. Deep Learning at a supply chain level shall impact lead time and on-time delivery.
  At a higher level of supply chain, producing only what the customer needs, when it needs it, in the required quality, the integration of deep learning technology will allow not only the systematic improvement of complex value chains, but a better use and exploitation of resources, thus reducing the environmental impact of industrial processes 4.0.
3. Deep Learning at a strategic level shall impact sustainable growth.
  At a more strategic level, customers and suppliers will be able to reach new levels of transparency and traceability on the quality and efficiency of the processes, which will generate new business opportunities for both, generating new products and services and cooperation opportunities in a cyber–physical environment. In a world of limited resources, increasing business volume can only be achieved by increasing the depth of integrated intelligence capable of successfully handling the emerging complexity in value streams.

To summarize, despite the "black box problem" and the challenge to have enough information and labeled data available for learning, Deep Learning will probably conquer in the field of machine vision, one country after another, and will act in the background without the user being aware of it. The role that Deep Learning will play in the creation of cyber–physical systems will be adopted from a strategic point of view, in which business leaders will tend to think of deep architectures as possible solutions to problems.

## Figures and Tables

**Figure 3:**
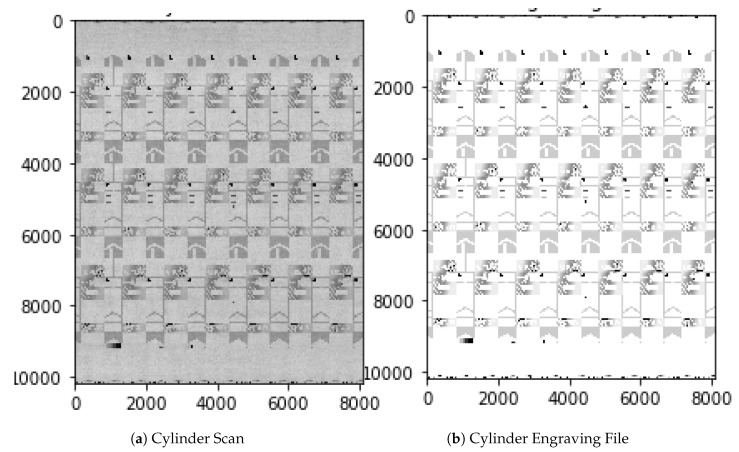
Cylinder Scan and Layout Engraving File.

**Figure 4:**
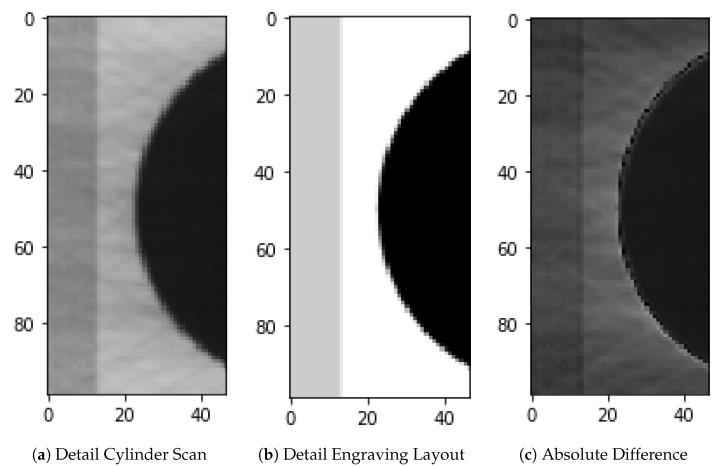
Example 1 of automatic selection of areas around possible errors.

**Table 1:** State of the Art.

Stage	Description of Improvement
Manual Inspection of Printed Product (Figure 2a)	In the first stage all cylinders of an order were printed together. Due to the processes used producing gravure cylinders, mistakes like holes in the cylinder are almost inevitable. To check the quality of the gravure cylinders, all the cylinders of one order are generally printed together and the resulting print checked manually with the help of a magnifying glass. To do this the approximate color of each individual cylinder must be mixed and all cylinders are printed one after the other on one substrate. On average this can be 5–10 cylinders or colours in one job. The big disadvantage is that all cylinders of a job must already be present. Thus, a one-piece flow is not possible. In addition, a lot of time is spent mixing the colours. As a direct comparison with the expected data was very difficult, the search for errors was focused on the most common errors that can happen during the production of an engraved printing cylinder. The coppering of the cylinder is a galvanic process, therefore it is possible that the cylinder has holes that also print. Another common mistake in the production of engraved printing cylinders is that parts that should print do not print. This can have different causes. Most of them can be traced back to problems during the engraving of the cylinder. To find these errors without a comparison to the expected data a search for irregularities in the carried out. As there are a lot of issues that had to be checked it was quite an ergonomically-challenging job, where some mistakes were not caught during the check.
Manual Inspection of Individual Color Printed Product (Figure 2b)	In the second stage the cylinders were all printed individually in the same (green) colour. In an attempt to further improve the quality control of each individual cylinder, the cylinder can also be printed itself. This impression was also checked manually with a magnifying glass by process experts. This has the advantage that there is no need to wait for the other cylinders of a job and no need to mix colours. However, the manual reading of the prints takes longer because there is one print for every cylinder of an order (5–10 cylinders) and not only one print for one order. Although this increased process reliability because process mistakes were directly tested on the product, the ergonomic weaknesses of the OQC process based on human experts could not be eliminated with this new improvement.
Evaluation of Errors by an Expert with aid of patented Software cLynx (Figure 2c)	This was then solved by the third stage: the digital scanning of the cylinder supported by the patented cLynx software (DE102017105704B3) [36]. To improve the quality and automate the process, a software named cLynx was developed to automatically compare the scanned file with the engraving file. The invention relates to a method for checking a printing form, in particular a gravure cylinder, for errors in an engraving printing form. A press proof of a cylinder gets printed and scanned using a high-resolution scanner. To compare the scans with the engraving file, a sequence of registration steps are performed. As a result the scans are matched with the engraving file. The differences between the two files are subject to a threshold in order to present the operator with a series of possible errors. As a result, the complexity of checking the entire print is reduced to a few possible errors that are checked by the operator. Since most of the work of troubleshooting was done by scanning + software, only the most conspicuous spots found by the software had to be evaluated by an expert.
Machine scans the cylinder and integrates the software cLynx (Figure 2d)	In the fourth stage, the entire printing process is omitted, as the cylinder surface is recorded directly with a camera within a cylinder scanning machine. To further reduce the cost of quality inspection, there is a need to check the cylinder without having to print it. To scan the surface of the cylinder a machine was built with a high-resolution line camera that scans the rotating cylinder at an approximate current speed of 1 meter/second. Because the scanning itself takes a minor portion of the processing time, this speed could actually be increased with a brighter LED lamp. After every movement a picture is taken, resulting in a flat image of the cylinder (Figure 3a). The main principles stay the same as with the scanned prints, as two complete recordings of the cylinder are made. These get matched to the engraving file and possible errors are presented to the operator using fixed thresholds (Figure 3b). This is done by automatically selecting areas around possible errors and calculating the absolute difference between the cylinder scan and the layout engraving file as shown in Figure 4. This significantly shortens the inspection time. However, the most prominent areas still have to be evaluated manually by the employee. For this reason, another fifth step towards a fully automated process is desired.

**Table 2:** DNN Architecture Detailed Description.

Layer Size	Layer Name	Layer Description and Rationale behind the Choice
(98, 98, 32)	conv2d 1 activation 1 (relu)	This is the first convolutional layer of the network. As observed in Figure 12 this layer mainly finds edges in the input image. In order to keep the values in check, an activation function is needed after each convolutional layer.
(49, 49, 32)	max pooling2d 1	In order to reduce the complexity of the convoluted result a max pooling layer is used. Only the maximum in this case of a 2 × 2 pixel window is chosen.
(47, 47, 64)	conv2d 2 activation 2 (relu)	In the second convolutional layer the results describe more complex forms as is visible in Figure 12. In order to keep the values in check, an activation function is needed after each convolutional layer.
(23, 23, 64)	max pooling2d 2	As with the previous max pooling layer this layer is used to reduce the complexity of the convoluted result.
(21, 21, 64)	conv2d 3 activation 3 (relu)	In the third convolutional layer resulting features are even more complex. In order to keep the values in check, an activation function is needed after each convolutional layer.
(10, 10, 64)	max pooling2d 3	As with the previous max pooling layer this layer is used to reduce the complexity of the convoluted result.
(8, 8, 32)	conv2d 4 activation 4 (relu)	This is the final convolutional layer with the most complex features. In order to keep the values in check, an activation function is needed after each convolutional layer.
(4, 4, 32)	max pooling2d 4	As with the previous max pooling layer this layer is used to reduce the complexity of the convoluted result.
(512)	flatten 1	The flatten layer is used to flatten the previous 3 dimensional tensor to 1 dimension.
(64)	dense 1 activation 5 (relu)	To further reduce the complexity we use a fully connected layer. Before the final connection takes place the relu function is used to zero out the negative results.
(1)	dense 2 activation 6 (sigmoid)	As the probability of the input image being an error is wanted, the sigmoid function is needed to transform the input value into a probability [0–1].

## Abbreviations

The following abbreviations are used in this manuscript:

IIoT	Industrial Internet of Things
OQC	Optical Quality Control
DNN	Deep Neural Networks
GPU	Graphic Process Unit
RAM	Random Access Memory
